# Thrombus molecular markers and thromboelastogram as early predictors of venous thromboembolism after total knee arthroplasty in perioperative elderly patients: A prospective study

**DOI:** 10.1097/MD.0000000000050228

**Published:** 2026-08-14

**Authors:** Bei Zhang, Huan Zhao, Jie Lu, Song Zhang, Li Chang

**Affiliations:** a Department of Laboratory Medicine, Hebei Medical University Third Hospital, Shijiazhuang, Hebei, People’s Republic of China; b Department of Rehabilitation, Hebei Medical University Third Hospital, Shijiazhuang, Hebei, People’s Republic of China.

**Keywords:** logistic regression, ROC, thromboelastography, thrombus molecular biomarkers, total knee arthroplasty

## Abstract

Elderly patients undergoing total knee arthroplasty have a higher incidence of venous thromboembolism (VTE) owing to age, surgical trauma, and other factors. Numerous studies have employed the Caprini scale and d-dimer levels to evaluate perioperative coagulation function and predict the occurrence of postoperative VTE. However, relatively few studies have assessed the risk of VTE by analyzing perioperative thrombus molecular markers and thromboelastography (TEG) findings in these patients. This prospective cohort study enrolled patients aged >60 years who underwent artificial knee replacement surgery between March 2024 and September 2024 at the Department of Arthroplasty of the Third Hospital of Hebei Medical University. The levels of thrombus molecular markers, including thrombin–antithrombin complex (TAT), plasmin-α_2_-plasmininhibitor complex, thrombomodulin, tissue plasminogen activator–inhibitor complex, and TEG, which included reaction time (R), clot kinetics (K), angle α, maximum amplitude, and clotting index (CI), were measured preoperatively and at postoperative hours 2 (poh2), day 1 (pod1), and day 3 (pod3). The patients were categorized into VTE and non-VTE groups based on color Doppler ultrasonography screening on postoperative day 4. We examined the disparities in general information indicators between the 2 groups, and logistic regression was used to analyze deep vein thrombosis risk factors in these patients. The diagnostic effectiveness of markers was determined using the receiver operating characteristic curve. TAT and TEG partial parameters are capable of reflecting the postoperative hypercoagulable state of the body with sensitivity; the combination of these parameters is anticipated to offer a strong predictive value in elderly patients. The median age in the VTE group was 71 (60–82), higher than 66 (60–83) in the non‑VTE group, and significant differences were found between the 2 groups (*P* = .025). Significant differences in R (*P* = .007), angle α (*P* = .029), and TAT (*P* = .047) on pod1 and clotting index (*P* = .027), TAT (*P* = .017), and TAT/plasmin inhibitor complex (*P* = .004) on pod3 were observed between the 2 groups. Binary logistic regression revealed that age, R value, angle α, TAT, and age were significant risk factors for deep vein thrombosis. Our research indicates that the area under the curve, sensitivity, and specificity of multi-index detection are superior to single-index detection.

## 1. Introduction

Total knee arthroplasty (TKA) is the preferred treatment for terminal knee disease and significantly improves patients’ quality of life and joint function.^[1]^ However, the postoperative hypercoagulable state predisposes patients to venous thromboembolism (VTE), a leading cause of perioperative mortality.^[2]^ VTE encompasses deep vein thrombosis (DVT) of the lower extremities and pulmonary embolism (PE), both of which pose serious health risks.^[3]^ The prevalence of DVT after major orthopedic surgery is 2.22% to 3.29% in Europe and the United States, and the prevalence of PE is 0.87% to 1.99%.^[4]^ The prevalence of DVT in Asian populations is 1.4%, and that of PE is 1.1%.^[5]^ One ultrasound screening study demonstrated that the prevalence of asymptomatic DVT after TKA was 47%.^[6]^ Once thrombosis occurs, it may delay rehabilitation and exercise, affecting postoperative knee function. In severe cases, it may even endanger a patient’s life.

Currently, several indicators including Caprini score, d-dimer, and P-selectin are utilized to evaluate coagulation status; however, none reliably predicts post-TKA thrombotic risk. In elderly TKA patients, Caprini, a general surgery static model, fails to reflect dynamic coagulation, lacks key surgical factors, and has poor sensitivity/specificity. d-dimer has low postoperative specificity, no unified cutoff, and cannot confirm/rule out thrombosis. P-selectin, a platelet/endothelial marker, is inflammation-affected, lacks standardized detection, and has insufficient predictive value.^[7–9]^ Factors such as plasmin inhibitor complex (PIC), thrombomodulin, thrombin–antithrombin complex (TAT), maximum amplitude, and clotting index (CI) can effectively predict the risk of DVT in patients with traumatic lower extremity fractures.^[10]^ Based on clinical experience, these indicators can also be used to predict the risk of venous thrombosis in elderly patients after total knee arthroplasty.

A small number of existing studies have explored the application of thromboelastography (TEG) for thrombosis screening after arthroplasty, while studies solely using thrombus molecular markers to predict postoperative thrombosis remain scarce. More importantly, no published literature has reported the combined detection of TEG and thrombus molecular biomarkers for the early prediction of venous thromboembolism in elderly patients following total knee arthroplasty. Accordingly, this prospective study addresses this research gap and aims to provide novel evidence for perioperative thrombotic risk evaluation in clinical practice.

## 2. Methods

This prospective observational cohort study was reported strictly in accordance with the Strengthening the Reporting of Observational Studies in Epidemiology guidelines for cohort studies.^[11]^ We plan to recruit patients, collect their baseline data, and detect thromboelastography (TEG) and the thrombus molecular biomarkers at different time points. Patients will be grouped based on the results of postoperative color Doppler ultrasound. Statistical analysis will be conducted on the data with significant differences between the 2 groups to identify sensitive indicators for thrombosis risk.

This study included patients aged 60 years and above who received TKA at the Department of Arthroplasty, Third Hospital of Hebei Medical University, between March 2024 and September 2024. All patients received standardized thromboprophylaxis care, including subcutaneous administration of low-molecular-weight heparin (enoxaparin sodium, 0.4 mL) 12 hours postoperatively; enoxaparin sodium was administered subcutaneously once daily at 0.4 mL before discharge. Patient selection was based on strict inclusion and exclusion criteria.

This prospective study strictly complies with the ethical principles for medical research involving human subjects laid down in the Declaration of Helsinki. The study protocol was approved by the Ethics Committee of the Third Hospital of Hebei Medical University (Approval No. W2023-066-1). The actual start date of the study was March 1, 2024, and the completion date was September 31, 2024. Participants were recruited and signed the written informed consent.

Patients were eligible for inclusion if they met the following criteria: negative preoperative color Doppler ultrasound of both lower extremities, age 60 years or older, and no history of previous knee surgery.

Patients were excluded if they had abnormalities in the coagulation or fibrinolytic system, had used antiplatelet and/or anticoagulant drugs within 1 week before surgery, or had hematologic disorders. Other exclusion criteria included intraoperative blood transfusion, hepatic or renal insufficiency, history of DVT, active infection, or malignant tumors. Patients were also excluded if preoperative color Doppler ultrasonography detected DVT in their lower extremities. In addition, patients with recent infections, trauma, or hemorrhage as well as individuals with uncontrolled diabetes mellitus or hypertension were not included in the study.

We adopted the single proportion formula to determine the sample size required for estimating the prevalence of hypercoagulability in patients with advanced malignant tumors. The single proportion formula is


$$n=\overset{2}{\underset{\frac{\alpha }{2}}{Z}}*\frac{p\left(1-p\right)}{d^{2}}$$

According to previous studies, the prevalence of hypercoagulability can reach 30%.^[12]^ Using this value (*P* = .30), with α = 0.05 (two-sided) and a margin of error *d* = 0.10, the formula yielded *n* ≈ 80. Considering potential dropouts and follow-up interruptions, we finally enrolled 100 patients to ensure a sufficient sample size and robust conclusions.

Based on our exclusion criteria, patients who were ultimately enrolled underwent bilateral lower extremity venous Doppler ultrasonography on postoperative day 4. According to the results of the ultrasound scan on day 4, all patients were divided into 2 groups: VTE and non-VTE groups. Patient demographic data, including age, sex, body mass index, comorbidities (e.g., hypertension), and history of prior joint replacement, were also collected. Surgical data such as operative time, anesthesia type, intraoperative blood loss, and transfusion details were also recorded.

The levels of thrombus molecular markers and TEG were measured preoperatively and at postoperative hours 2 (poh2), day 1 (pod1), and day 3 (pod3).

## 3. Laboratory evaluations and diagnostic methods

Venous blood samples were collected preoperatively and on poh2, pod1, and pod3 for thrombus molecular biomarkers and TEG analysis. All patients underwent preoperative Doppler ultrasonography to exclude preexisting DVT. Biomarker detection was performed using a fully automated chemiluminescence immunoassay analyzer (Sysmex HISCL5000; Sysmex Corporation) and an elastography detector (TEG200; Haemonetics Corporation). The evaluated indices included TEG parameters consisting of the R value, K value, angle α, maximum amplitude value, lysis at 30 min, estimated percentage of lysis, and CI index, and thrombus molecular markers composed of TAT, PIC, tissue plasminogen activator–inhibitor complex, and thrombomodulin.

## 4. Statistical analysis

We identified data showing significant differences in the test values of indicators and baseline characteristics between VTE and non-VTE patients at different time points and incorporated such data into a multivariate model.

Data analyses were performed using the SPSS statistical software (version 26.0; SPSS Inc.). Counting data were expressed as percentages, and the chi-square test was used for categorical variables. For continuous variables, differences between groups were analyzed using a Student *t* test or Mann–Whitney *U* test, depending on the normality of the data. Shapiro–Wilk test was performed to assess the normality of continuous variables. For normally distributed data with homogeneous variance, Student *t* test was used for intergroup comparisons; non-normally distributed data were analyzed via the Mann–Whitney *U* test (nonparametric test). Categorical variables were compared using the chi-square test. Binary logistic regression analysis was conducted to calculate the risk factors for VTE after TKA. The diagnostic efficacy of each index was evaluated using the receiver operating characteristic (ROC) curve, and the area under the curve (AUC), sensitivity, specificity, and cutoff values were calculated. An AUC ≥ 0.9 indicated a higher predictive value, 0.7 ≤ AUC < 0.9 indicated a moderate predictive value, and an AUC < 0.7 indicated a low predictive value. Statistical significance was set at *P* < .05.

## 5. Results

**Figure 1.**
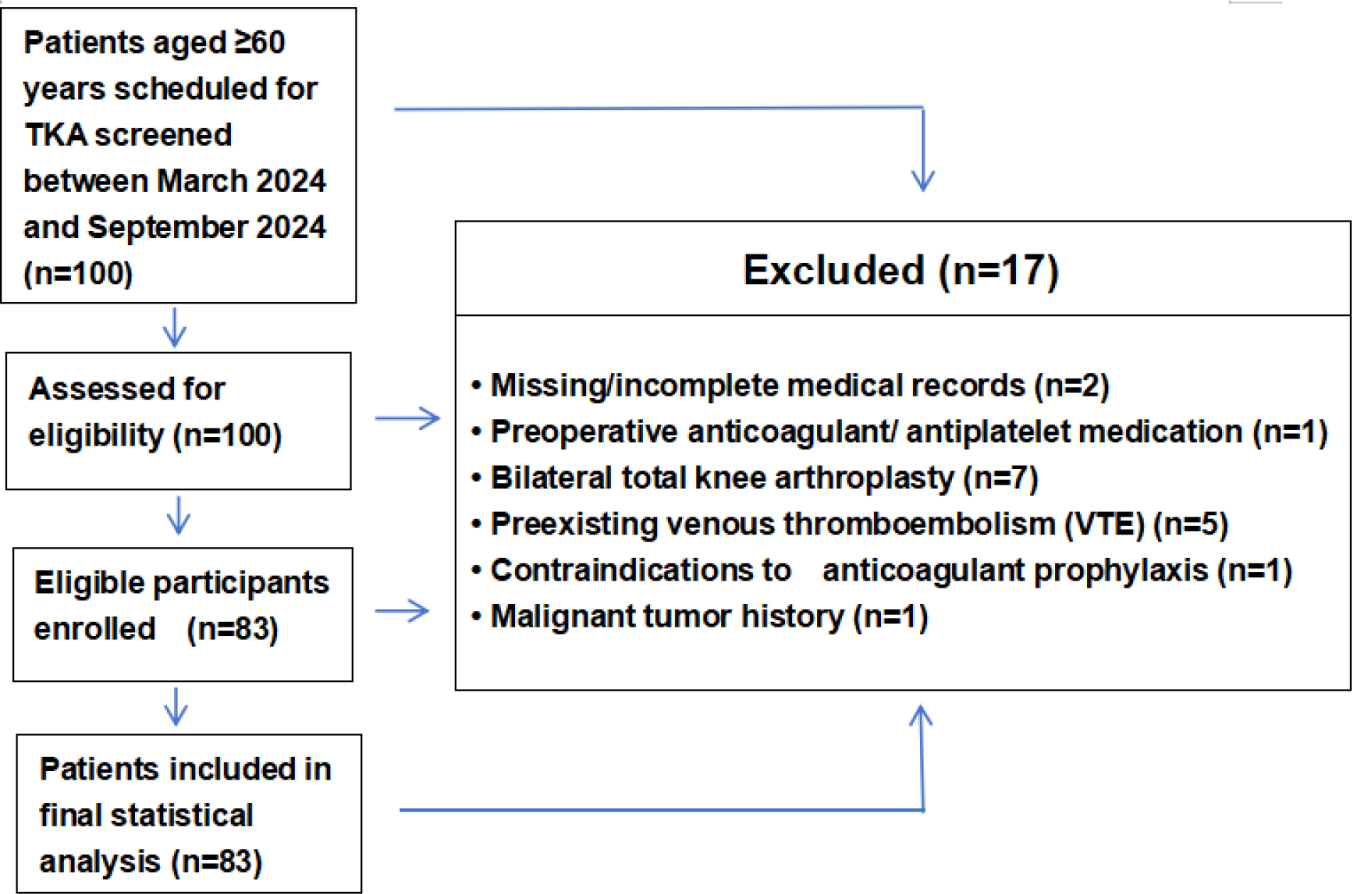
Exclusion criteria and the number of studies included in this study. TKA = total knee arthroplasty.

The presence of VTE in 25 of the 83 patients was confirmed by ultrasound examination 4 days after surgery; the incidence of VTE is 30.1%. Detailed information is shown in Figure 1. There were 2 males and 23 females among the 25 VTE patients, including 2 with posterior tibial vein thrombosis and 1 with peroneal vein thrombosis, all of whom were female, and the rest had intermuscular vein thrombosis.

As shown in Table 1, the median age of patients in the VTE group was 71 (60–82) compared with 66 (60–83) in the non‑VTE group, the mean age of the patients in the VTE group was significantly higher than that of the non-VTE group and was statistically significant (*P* = .025), while other indicators showed no significant difference between the 2 groups.

**Table 1:** Comparison of clinical data between the VTE group and non-VTE group n (%).

Variable	VTE (n = 25)	Non-VTE (n = 58)	*x^2^/ Z *	*P* < .05
Age (yr)	71 (60, 82)	66 (60, 83)	2.244	.025*
Height (m)	1.60 (1.52, 1.73)	1.60 (1.48, 1.83)	0.878	.380
Weight (kg)	70 (46, 90)	70 (50, 95)	0.199	.842
BMI	27.12 ± 3.12	26.95 ± 3.33	0.804	.421
Surgical duration (min)	85 (60, 190)	90 (65, 254)	0.652	.514
Anesthesia duration (min)	150 (100, 230)	150 (105, 300)	0.928	.353
Intraoperative bleeding (mL)	120 (50, 200)	100 (50, 150)	0.863	.388
Gender			6.939	.008
Male	2/23	21/23		
Female	23/60	37/60		
Anesthesia method			0.000	.986
General anesthesia	16/53	37/53		
Epidural anesthesia	9/30	21/30	0.012	
ASA grade				.913
Ⅱ grade	20/67	47/67		
>Ⅱ grade	5/16	11/16		

Data are presented as mean ± standard deviation or quartiles. The Mann–Whitney *U* test was used for intergroup comparison of continuous variables, and the chi-square test was applied for categorical variables.

ASA = American Society of Anesthesiologists, BMI = body mass index, VTE = venous thromboembolism.

* *P* < .05.

As shown in Table 2, there was no statistically significant difference in any of the indices of TEG and thrombus molecular markers preoperatively and at poh2. Significant differences were observed in the R value (*P* = .007), angle α (*P* = .029), and TAT (*P* = .047) of the thrombus molecular markers in the 2 groups on pod1. Significant differences were observed in the CI index of the TEG (*P* = .027), TAT (*P* = .017), and TAT/PIC (*P* = .004) of the thrombus molecular markers on pod3.

**Table 2:** Differences between the VTE group and the non-VTE group at preoperative, poh2, pod1, and pod3.

Variable	Preoperation		*Z*	*P*	Poh2		*Z*	*P*	Pod1		*Z*	*P*	Pod3		*Z*	*P*
	Non-VTE group	VTE group			Non-VTE group	VTE group			Non-VTE group	VTE group			Non-VTE group	VTE group		
R	6.10 (5.50, 7.00)	5.80 (5.35, 7.00)	0.586	.558	4.80 (4.40, 5.20)	4.70 (4.15, 5.00)	1.990	.052	5.20 (4.50, 5.88)	4.70 (4.20, 5.20)	2.683	.007**	5.20 (4.90, 5.90)	1.30 (1.10, 1.50)	1.804	.071
*K*	1.70 (1.40, 2.00)	1.60 (1.35, 1.95)	0.726	.458	1.80 (1.48, 2.20)	1.60 (1.25, 2.00)	1.656	.098	1.70 (1.30, 2.20)	1.50 (1.25, 1.85)	1.085	.278	1.35 (1.20, 1.60)	1.30 (1.10, 1.50)	1.249	.212
α	66.20 (63.90, 69.50)	67.30 (65.15, 70.35)	0.815	.415	65.95 (61.10, 69.40)	68.90 (64.00, 71.90)	1.985	.057	66.30 (60.85, 70.00)	69.70 (65.30, 72.00)	2.184	.029*	70.25 (66.75, 72.43)	71.80 (69.75, 74.15)	1.787	.074
MA	65.00 (61.60, 68.20)	65.00 (61.40, 68.50)	0.164	.870	61.65 (56.20, 65.60)	64.40 (58.35, 66.90)	1.325	.185	63.65 (58.78, 67.13)	63.90 (62.05, 68.40)	1.315	.188	67.05 (63.55, 71.23)	71.80 (69.75, 74.15)	0.953	.341
LY30	0.25 (0.00, 1.00)	0.30 (0.00, 1.00)	0.050	.960	0.00 (0.00–0.10)	0.00 (0.00–0.10)	1.022	.307	0.30 (0.00, 1.33)	0.80 (0.00, 1.50)	0.505	.613	0.90 (0.45, 1.93)	0.80 (0.00, 1.50)	0.730	.465
EPL	0.25 (0.00, 1.00)	0.30 (0.00, 1.00)	0.050	.960	0.00 (0.00–0.10)	0.00 (0.00–0.10)	0.955	.340	0.35 (0.00, 1.33)	0.80 (0.00, 1.50)	0.657	.511	1.50 (0.675, 2.75)	1.60 (0.60, 2.65)	0.268	.789
CI	0.55 (–0.60, 1.00)	0.50 (–0.15, 1.40)	0.522	.602	1.0 (–0.20,1.60)	1.50 (0.55, 2.75)	1.887	.059	1.00 (–0.60, 1.73)	1.70 (0.80, 2.95)	2.403	.056	1.75 (0.875, 2.70)	2.30 (1.50, 3.30)	2.211	.027*
TAT	2.30 (1.70, 3.70)	2.20 (1.30, 3.65)	0.581	.561	25.45 (15.40, 33.40)	30.80 (18.80, 38.90)	1.156	.248	14.45 (9.48, 19.68)	15.80 (13.10, 25.60)	1.990	.047*	9.10 (6.68, 12.10)	11.60 (8.90, 16.20)	2.393	.017*
PIC	0.42 (0.33, 0.49)	0.43 (0.35, 0.61)	0.606	.545	2.12 (1.49, 2.82)	2.20 (1.43, 2.86)	0.119	.905	1.18 (0.75, 1.84)	1.23 (0.90, 1.51)	0.665	.506	0.75 (0.65, 0.93)	0.73 (0.53, 0.97)	0.804	.421
TM	9.90 (7.0, 11.95)	7.0 (6.00, 11.10)	2.08	.057	5.65 (4.90, 8.60)	5.40 (4.35, 6.15)	1.360	.174	6.30 (5.50, 8.43)	6.00 (5.05, 7.50)	1.142	.253	6.90 (5.90, 10.10)	6.70 (5.95, 8.60)	0.506	.613
4	5.75 (4.20, 7.45)	5.70 (4.15, 6.20)	1.574	.115	5.40 (4.35, 6.15)	5.50 (4.65,7.25)	0.417	.677	5.75 (4.30, 6.90)	5.10 (4.60, 6.50)	0.020	.984	4.25 (3.10, 5.23)	4.60 (4.35, 6.05)	2.036	.042
TAT/PIC	5.70 (4.10, 7.80)	4.90 (4.13, 6.58)	0.888	.374	10.69 (8.79, 14.52)	12.25 (10.06, 16.48)	1.	.177	12.44 (9.55, 15.38)	15.19 (10.55, 20.01)	1.658	.097	11.50 (8.13, 15.26)	16.19 (11.94, 21.61)	2.849	.004**

Data are presented as mean ± standard deviation or quartiles. The Mann–Whitney *U* test was used for intergroup comparison of continuous variables, and the chi-square test was applied for categorical variables.

α = angle α, CI = clotting index, EPL = estimated percentage of lysis, K = clot kinetics, LY30 = lysis at 30 min, MA = maximum amplitude, PIC = plasmin inhibitor complex, poh2 = postoperation day 2, pod1 = postoperation day 1, and pod3 = postoperation day 3, R = reaction time, TAT = thrombin–antithrombin complex, TM = thrombomodulin, t-PAIC = tissue plasminogen activator–inhibitor complex, VTE = venous thromboembolism.

* *P* < .05.

** *P* < .01.

Binary logistic regression analysis was conducted on the indicators of statistical significance on pod1, taking the occurrence of VTE as the dependent variable and the indices showing statistical differences between the groups as covariates. Age (odds ratio [OR] = 1.121; 95% confidence interval [95% CI]: 1.003–1.252; *P* = .043), R value (OR = 0.310; 95% CI: 0.112–0.859; *P* = .024), angle α (OR = 1.162; 95% CI: 1.027–1.314; *P* = .017), and TAT (OR = 1.121; 95% CI: 1.022–1.229; *P* = .015) were risk factors for VTE in TKA on pod1. Please refer to Table 3 for further details.

**Table 3:** Logistic regression analysis of influencing factors of VTE on pod1 formation in TKA.

Variable	B	Standard error	Wald	OR	95% CI	*P*
Age	.114	0.57	4.080	1.121	1.003–1.252	.043
R value	−1.170	0.519	5.0720	0.310	0.112–0.859	.024
Angle α	.150	0.063	5.712	1.162	1.027–1.314	.017
TAT	.114	0.047	5.866	1.121	1.022-1.229	.015

95% CI = 95% confidence interval, OR = odds ratio, R = reaction time, pod1 =  postoperative day 1, TAT = thrombin–antithrombin, TKA =  VTE =  venous thromboembolism.

**Figure 2.**
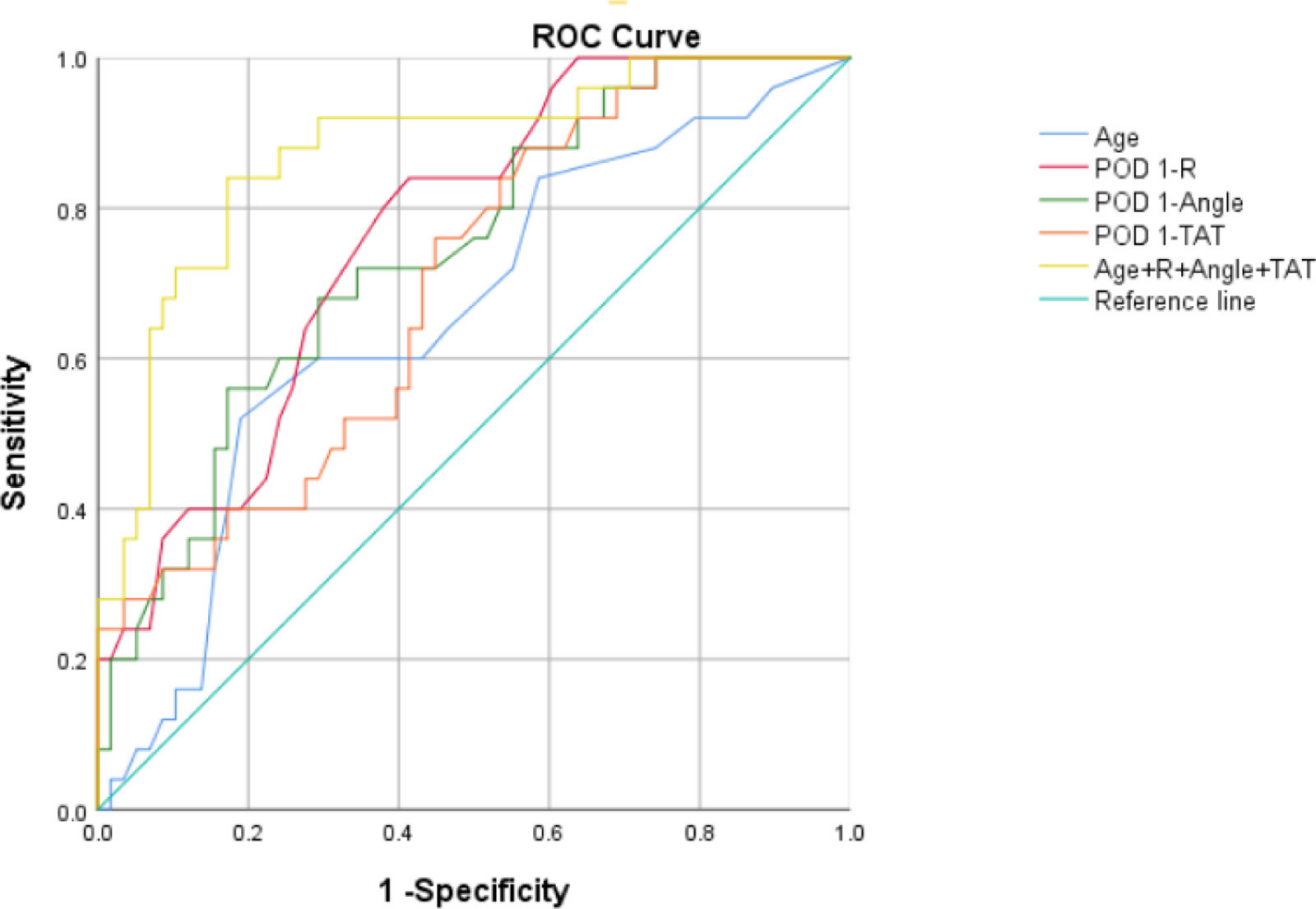
Age, R value, angle α, TAT, and combined indicators to predict the ROC curve of VTE. POD 1 = postoperative day 1, R = reaction time, ROC = receiver operating characteristic curve, TAT = thrombin-antithrombin complex, VTE = venous thromboembolism.

Efficacy analysis of thrombus molecular markers and thromboelastograms for predicting DVT in TKA was conducted using ROC curve analysis. The results indicated that age, Pod 1R, Pod 1-Angleα, and Pod 1-TAT demonstrated high diagnostic values, with AUC values of 0.656 (95% CI: 0.527–0.784; *P* = .025), 0.766 (95% CI: 0.662–0.869; *P* = .07), 0.740 (95% CI: 0.0.627–0.853; *P* = .029), and 0.699 (95% CI: 0.581–0.816; *P* = .047), respectively. The sensitivity and specificity were 52, 96, 96, 88%, and 81%, 40%, 66%, 59% of the above indicators, respectively. The combined diagnostic approach using age, Pod 1R, Pod 1-Angleα, and Pod 1-TAT for VTE had an AUC of 0.875 (95% CI: 0.791–0.959; *P* = .001), with sensitivity and specificity rates of 0.92 and 0.55, respectively. Please refer to Table 4 and Figure 2 for the detailed data. In addition, Cox and Snell *R*-square and Nagelkerke *R*-square were analyzed to quantitatively evaluate the goodness-of-fit effect. The Cox and Snell *R*-square based on logistic regression analysis was 0.421, and the Nagelkerke *R*-square was 0.647.

**Table 4:** VTE efficacy analysis of both single and combined indices in the diagnosis of TKA index.

Variable	Sensitivity (%)	Specificity (%)	AUC	95% CI	*P*	Cutoff value
Age	52	81	0.656	0.527–0.784	.025	70.5
Pod 1-R	96	40	0.766	0.662–0.869	.07	5.5
Pod 1-Angle α	96	66	0.740	0.627–0.853	.029	61.25
Pod 1-TAT	88	59	0.699	0.581–0.816	.047	9.65
Age + R + Angle α+TAT	92	55	0.875	0.791–0.959	.001	–

95% CI = 95% confidence interval, AUC = area under the curve, Pod = postoperative day, R = reaction time, TAT = thrombin–antithrombin, TKA = total knee arthroplasty, VTE =  venous thromboembolism.

## 6. Discussion

In our study, the incidence of VTE after TKA was observed to be 30.12% (25/83 cases), including 2 cases of posterior tibial vein thrombosis, 1 case of peroneal vein thrombosis, and the rest being intermuscular vein thrombosis. VTE remains a serious clinical complication after TKA.^[13,14]^ The surgical process can lead to coagulation disorders in patients. During surgery, the use of anesthetics can cause vasodilation and a decrease in the contraction of the lower-limb muscles, which results in slow blood flow in the vessels. All these factors can increase the incidence of VTE.^[15,16]^ Evtugina et al^[17]^ reported that the incidence of postoperative DVT in surgical patients without prophylactic measures could be up to 10%. We found that the incidence of VTE remained as high as 30.12% despite preventive measures.

As shown in Table 1, there was a significant difference in age between the VTE and non-VTE groups. Studies have shown that geriatric hip fracture patients often experience significant self-care impairment. Only a small proportion of patients can regain preinjury ambulatory function.^[18,19]^ Early initiation of thromboprophylaxis is crucial in geriatric patients with multiple risk factors. Equally important is an accurate assessment of thrombotic-complication probability. This can guide the clinical selection of appropriate anticoagulants and mechanical thromboprophylaxis, effectively reducing the risk of VTE.^[20]^ Therefore, it is necessary to identify high-risk patients who may develop VTE using certain methods and further explore the necessity of enhancing VTE prevention measures. This is the significance of our study.

TEG has been extensively applied for the risk assessment of thrombophilia. The principle of TEG is to simulate the human venous environment and dynamically and real-timely capture the coagulation process via receptor-driven curves. TEG directly reflects the dynamic evolution of blood coagulation, including the rate of thrombus formation, the rigidity of thrombi, and the activity of the fibrinolytic system. The results of an observational study suggest that a decrease in the R value indicates that the patient’s blood tends to be hypercoagulable with an elevated risk of DVT.^[21]^ The *K* value has been demonstrated to be a sensitive indicator of changes in fibrinogen concentration in elderly patients with bone fractures. A decrease in this value implies that blood is prone to hypercoagulability.^[22]^ An elevated TAT level signifies substantial generation and enhanced activity of thrombin, indicating activation of the coagulation system.^[23]^ PIC can effectively reflect the fibrinolytic state of an organism, with its main physiological function being the degradation and solubilization of fibrin in blood vessels.^[24]^ Given that the balance between the fibrinolytic and coagulation systems is strictly regulated, once the coagulation system is activated, the fibrinolytic system is promptly activated. A higher PIC level indicates stronger fibrinolytic activity and a greater risk of bleeding, which also provides evidence of the occurrence of thrombosis.^[25]^ As shown in Table 2, none of the indicators showed significant differences between the 2 groups preoperatively and at poh2. This may be because the stress reaction of the body triggered by the surgical trauma had not yet fully manifested at this time. In contrast, on pod1, significant differences were found in the R value, angle α, and CI index of TEG, as well as TAT among the thrombus molecular markers between the 2 groups. These changes may be closely related to the rapid dynamic adjustment of the coagulation and fibrinolytic systems in the early postoperative period. Therefore, timely detection of these indicators is of clinical significance for the early and accurate assessment of thrombosis risk. On pod3, the CI index, TAT, and TAT/PIC still showed statistical significance, suggesting that these indices could still indicate a thrombotic tendency in patients during the middle stage of postoperative recovery. Previous research has indicated the level of TAT may be associated with the incidence of VTE.^[26]^ Consistent with this, Kobayashi et al.^[27]^ reported significant differences in TAT levels between the DVT group and the normal group after medial opening wedge high tibial osteotomy (*P* < .05, 1 report; *P* < .001). However, few studies have used the TAT to assess the risk of VTE after TKA. In our study, we found significant differences in TAT levels between the 2 groups on pod1 and pod3. Since lots of thrombosis occurred earlier and there were more significant differences than at other time points, we observed differences between the 2 groups after surgery. We analyzed the data on pod1. Binary logistic regression analysis identified age, R value, angle α, and TAT as risk factors for VTE in patients who underwent TKA. The diagnostic capabilities of age, R value, angle α, TAT, and their combination were assessed through ROC curve analysis, revealing that these markers had strong diagnostic performance with AUC values for VTE diagnosis at 0.656, 0.766, 0.740, 0.699, and 0.875, respectively. These indicators have the disadvantage of good sensitivity but poor specificity, and the specificity of the evaluation can be improved by increasing the sample size.

E Jianfei et al.^[28]^ found that TEG and thrombotic molecular markers showed strong diagnostic efficacy in the early detection of DVT associated with traumatic lower extremity fractures, and there was no significant difference in their diagnostic performance. Therefore, TEG and thrombotic molecular markers have powerful clinical diagnostic capabilities in the early detection of DVT and present great potential in clinical practice. However, our study has some limitations. First, this was a single-center, prospective study. Second, the sample size is relatively small. Multicenter prospective studies and large sample clinical studies are needed to illustrate and predict DVT in the future. In summary, this study demonstrated that TEG and thrombus biomarkers have a high sensitivity but low specificity for the prediction of venous thrombosis.

These results suggest that the levels of age, R, angle α, and TAT on pod1 may predict the occurrence of VTE. This study indicated that single-indicator prediction is inferior to combined-indicator prediction. Future studies are needed to identify more sensitive indicators for determining the risk of VTE.

## Author contributions

**Data curation:** Bei Zhang.

**Funding acquisition:** Bei Zhang.

**Writing – original draft:** Bei Zhang.

**Investigation:** Huan Zhao.

**Software:** Huan Zhao, Jie Lu.

**Supervision:** Huan Zhao.

**Formal analysis:** Jie Lu.

**Validation:** Jie Lu.

**Methodology:** Song Zhang.

**Visualization:** Song Zhang.

**Writing – review & editing:** Song Zhang, Li Chang.

**Conceptualization:** Li Chang.

**Project administration:** Li Chang.

**Resources:** Li Chang.
